# Editorial for the Special Issue Energy Conversion and Storage Devices: Materials and Applications

**DOI:** 10.3390/mi16080843

**Published:** 2025-07-23

**Authors:** Tejaswi Tanaji Salunkhe, Il Tae Kim

**Affiliations:** School of Chemical, Biological & Battery Engineering, Gachon University, Seongnam 13120, Republic of Korea

The increasing demand for high-performance portable electronics, electric vehicles (EVs), grid-scale storage, and sustainable energy systems is driving transformative progress in the field of energy conversion and storage technologies. As societies worldwide work toward achieving carbon neutrality and meeting the sustainable development goals, a pressing need for energy devices that offer not only higher energy and power densities but also enhanced safety, cost-effectiveness, environmental compatibility, and long-term durability is emerging. These evolving demands have stimulated extensive research into advanced materials design, novel electrode and electrolyte chemistries, and smart device architectures across a wide range of energy storage and conversion platforms, including batteries, supercapacitors, fuel cells, nanogenerators, and hybrid systems [1,2,3].

This Special Issue of *Micromachines*, “Energy Conversion and Storage Devices: Materials and Applications,” serves as a focused platform for recent advances and breakthroughs in this multidisciplinary field. It is a collection of original research articles and reviews exploring innovative approaches in material synthesis, micro-/nano-structuring, scalable fabrication techniques, and integrated system design. The contributions span diverse technologies and highlight both fundamental insights and practical solutions with the potential to drive the development of next-generation sustainable and efficient energy systems. Collectively, this issue reflects the critical roles that materials science, electrochemistry, nanotechnology, and microsystems engineering play in shaping the future of energy [2,3,4,5].

Working in the realm of supercapacitor research, Gaikwad et al. [contribution 1] presented a novel 3D flower-like quaternary CuNiCoZnO architecture developed on multiple substrates. Their work provides a promising strategy for achieving high specific capacitance and cycling stability. Complementarily, Mane et al. [contribution 2] synthesized low-dimensional ZnS nanoparticle clusters via a simple one-pot method. Their electrodes exhibited excellent electrochemical properties, making them suitable for future energy storage systems. Both of these contributions significantly enrich the field of high-performance supercapacitor development through scalable and cost-effective materials synthesis techniques.

From a battery technology perspective, Salunkhe and Kim [contribution 3] introduced expanded graphite as a high-efficiency anion host for lithium dual-ion batteries (Li-DIBs), achieving a remarkable output voltage of 4.62 V and enhanced energy density. Their study provides insights into the structural engineering of carbon-based materials for next-generation battery applications. This work is also supported by its high citation impact and leadership.

Exploring the interplay between hydration, temperature, and ionic conductivity, Zărnescu et al. [contribution 4] investigated Na_x_CoO_2_-based transducers. Their work offers valuable implications for sensor applications and sodium-based electrochemical systems by revealing how thermal and moisture variations influence voltage responses. Such research contributes to a broader understanding of electrolyte behavior in alternative energy conversion technologies.

Focusing on innovative energy-harvesting technologies, Cai et al. [contribution 5] reported a U-shaped tube-based liquid–solid triboelectric nanogenerator designed to capture unused compressed air energy. This approach is notable for its creativity and practical application in industrial settings. Likewise, Kim et al. [contribution 6] developed a hybrid 1D–2D non-contact mode triboelectric sensor capable of detecting string instrument frequencies. Although it is primarily targeted at acoustic sensing, their work introduces novel transduction techniques with potential crossover into wearable and self-powered electronic devices.

Finally, Hornik et al. [contribution 7] provided experimental proof of concept for 3D-printed microfluidic benthic microbial fuel cells (MBMFCs) incorporating carbon-fiber electrodes. This pioneering development bridges microfluidics, additive manufacturing, and microbial fuel cells to create compact, biocompatible, and efficient power sources suitable for aquatic and environmental monitoring applications.

Collectively, these contributions illustrate a dynamic convergence of materials science, nanotechnology, electrochemistry, and microengineering. This Special Issue reflects the rapid strides made in energy research and serves as a valuable resource for scientists, engineers, and practitioners striving to discover cleaner and more efficient energy solutions.

We extend our sincere gratitude to all authors for their impactful contributions and to the reviewers for their invaluable insights. We also acknowledge the editorial support team at *Micromachines* for facilitating the publication of this Special Issue.
